# Dietary Protein Considerations to Support Active Aging

**DOI:** 10.1007/s40279-014-0258-7

**Published:** 2014-10-30

**Authors:** Benjamin T. Wall, Naomi M. Cermak, Luc J. C. van Loon

**Affiliations:** 1 NUTRIM School for Nutrition, Toxicology and Metabolism, Maastricht University, Maastricht, 6200 MD The Netherlands; 2 Department of Human Movement Sciences, Maastricht University, Maastricht, 6200 MD The Netherlands

## Abstract

Given our rapidly aging world-wide population, the loss of skeletal muscle mass with healthy aging (sarcopenia) represents an important societal and public health concern. Maintaining or adopting an active lifestyle alleviates age-related muscle loss to a certain extent. Over time, even small losses of muscle tissue can hinder the ability to maintain an active lifestyle and, as such, contribute to the development of frailty and metabolic disease. Considerable research focus has addressed the application of dietary protein supplementation to support exercise-induced gains in muscle mass in younger individuals. In contrast, the role of dietary protein in supporting the maintenance (or gain) of skeletal muscle mass in active older persons has received less attention. Older individuals display a blunted muscle protein synthetic response to dietary protein ingestion. However, this reduced anabolic response can largely be overcome when physical activity is performed in close temporal proximity to protein consumption. Moreover, recent evidence has helped elucidate the optimal type and amount of dietary protein that should be ingested by the older adult throughout the day in order to maximize the skeletal muscle adaptive response to physical activity. Evidence demonstrates that when these principles are adhered to, muscle maintenance or hypertrophy over prolonged periods can be further augmented in active older persons. The present review outlines the current understanding of the role that dietary protein occupies in the lifestyle of active older adults as a means to increase skeletal muscle mass, strength and function, and thus support healthier aging.

## Progressive Muscle Loss in the Older Adult

Global demographics indicate that the number of individuals aged 60 years and over is set to triple by the year 2050, with the fastest growing sub-population being those aged over 85 years [1]. A key hallmark of aging is a progressive loss of muscle mass, which occurs independently of health status [2]. The association between muscle loss and increased incidence of falls, fractures, metabolic disease and other health complications, indicates that the burden of our aging society on healthcare systems will increase dramatically over the upcoming decades [1, 2]. The physiological mechanisms underpinning the age-related loss of muscle mass and strength are complex and multifactorial, and remain to be fully elucidated. It has been established that reduced levels of physical activity, prevalence of disease, periods of hospitalization, and an inadequate diet all play a role in this process [2]. However, it should be noted that muscle tissue of adults of all ages and health statuses retains the ability to recondition in response to regular exercise, either with respect to muscle hypertrophy or an increase in oxidative capacity [3–9]. Moreover, it is clear that those older adults who maintain high physical activity levels throughout their lifespan experience significantly less muscle loss compared with their more sedentary peers [3–9]. Importantly, data generated thus far indicates that resistance-type exercise training yields the clearest benefits with respect to maintaining (or increasing) muscle mass as we age [3–9]. In accordance with the positive effects of active aging, reports of the magnitude of age-related muscle loss vary greatly from ~0.5 to 2 % of total muscle mass loss per year from the age of ~50 years onwards [10–12]. A large proportion of this variance can indeed be explained by the habitual physical activity levels and/or training status of the individuals studied [9]. As such, the importance of retaining an active lifestyle to support healthy aging is unequivocal. However, even the active older adult still experiences some muscle loss with advancing age. Even modest losses of muscle mass over the advancing years will compromise strength, oxidative capacity and overall exercise performance [2, 9, 13–15], and may hinder the ability to remain active, thus instigating a vicious circle of declining health. As such, muscle loss with aging should not only be viewed as a clinical challenge to the most frail, sarcopenic elderly [16], but also as a concern for those elderly wishing to stay (or become more) physically active in an effort to improve their quality of life.

Optimal nutrition has long been considered a key consideration for maintaining a healthy lifestyle, and the benefits of correctly applied nutritional support for young individuals involved in regular training have been studied extensively [17–19]. Of particular relevance, the amount, type and timing of dietary protein consumed during prolonged resistance-type exercise training has been shown to modulate gains in muscle mass and strength in young men [17, 18, 20–24]. In contrast, however, less research has been directed at how nutrition can be used to maximize the skeletal muscle adaptive response to a more physically active lifestyle in the older population. The present review will discuss muscle protein metabolism in elderly individuals and address the key considerations relating to dietary protein intake to support healthy aging in the active older adult.

## Nutritional Regulation of Muscle Protein Metabolism in the Elderly

From a physiological standpoint, skeletal muscle mass is in a constant state of turnover, with muscle proteins being synthesized and broken down simultaneously throughout the day. Accordingly, relevant gains or losses of skeletal muscle mass must be attributed to a persistent alteration in muscle protein synthesis rates, breakdown rates, or a combination of the two. Daily muscle protein turnover (~1–2 %) is regulated in large part by nutrition [25]. Specifically, protein and/or amino acid intake increases muscle protein synthesis rates (and also inhibits muscle protein breakdown rates to a lesser extent), thereby allowing net muscle protein accretion. The post-prandial elevation in muscle protein synthesis rates are driven by the rise in plasma concentration of the essential amino acids [26], and leucine in particular [27, 28], whereas the inhibition of protein breakdown is mainly attributed to hyperinsulinemia [29, 30]. These post-prandial periods offset the net loss of muscle protein which occurs during fasting periods. Consequently, both post-absorptive muscle protein turnover rates as well as the magnitude of post-prandial stimulation of muscle protein synthesis rates are viewed as key factors for skeletal muscle mass maintenance.

Attempts to elucidate the impairments in muscle protein metabolism that may underpin age-related muscle loss initially focused on assessing basal (post-absorptive) muscle protein synthesis and/or breakdown rates in young and elderly subjects. Early studies observed no age-related differences in basal muscle protein breakdown rates [9], but found considerably lower mixed, myofibrilar, and/or mitochondrial muscle protein synthesis rates in older compared with younger men [31–35]. However, more recent studies have been unable to reproduce these findings, with no measurable differences being reported between older and younger men [28, 36–42]. The apparent discrepancy may be attributed to differences in study design and health status, habitual physical activity level and/or dietary habits between the recruited cohorts of subjects [36, 43]. Due to this uncertainty, research focus has since shifted to the assessment of the impact of aging on potential impairments in the muscle protein synthetic response to the main anabolic stimuli: food intake and physical activity.

Much data has accumulated in recent years demonstrating that elderly individuals have a blunted muscle protein synthetic response to either intravenous [41, 44] or oral [38, 39] administration of essential amino acids. An interesting additional consideration is that some data also suggest that the normal post-prandial inhibition of muscle protein breakdown (primarily due to the rise in circulating insulin) may be blunted in elderly men [45]. Collectively, this reduced responsiveness to meal ingestion in older adults has been termed ‘anabolic resistance’ and is now commonly thought to be a key factor in the etiology of sarcopenia [46–49]. However, the mechanisms underlying anabolic resistance remain to be established. It is logical that impairments may reside at the level of protein digestion [50–52], amino acid absorption [50–52], the post-prandial hormonal response and subsequent microvascular perfusion [53, 54], amino acid uptake in skeletal muscle tissue [55], intramuscular signaling [38, 56], and/or myofibrillar muscle protein accretion [38]. Impaired dietary protein digestion and subsequent amino acid absorption rates and/or a greater retention of ingested amino acids within the splanchnic area have been observed in older compared with younger men [42, 57], although not all studies confirm this [58]. Such findings imply that fewer amino acids may become available for post-prandial muscle protein synthesis. Evidence also exists suggesting that insulin resistance at the level of capillary recruitment-mediated muscle perfusion following meal ingestion is a key factor underlying anabolic resistance [53, 54, 59]. Some data demonstrate a reduced gene and/or protein expression of key muscle amino acid transporter proteins in older men following meal ingestion [60]. However, dynamic measurements of intracellular amino acid transport in young and older men following meal ingestion are necessary to confirm whether this is a potential site of regulation for age-related anabolic resistance. The intracellular signaling pathways responsible for regulating muscle protein synthesis are highly dependent upon the activation of the mammalian target of rapamycin (mTOR) [61, 62]. Indeed, mTOR signaling is highly responsive to an increase in plasma and/or intracellular essential amino acid concentrations, with leucine being of particular relevance [63]. It has been shown that the activation (in response to amino acids and/or insulin) of mTOR and its key downstream targets controlling translation initiation (e.g., P70S6K) is impaired with aging [38, 44]. Though a reduced ability of the senescent muscle cell to sense and/or transduce a nutrient signal may be a mechanistic basis for anabolic resistance, this could also simply be a consequence of reduced amino acid delivery by any of the previously mentioned potential sites of (dys)regulation.

Overall, it is clear that anabolic resistance may be explained by impairments at various levels of physiology. However, of specific interest is the observation that physical activity performed prior to the ingestion of a meal-like amount of dietary protein can compensate for anabolic resistance in the older adult, resulting in muscle protein synthesis rates not different from those observed in the young [52]. Physical activity sensitizes skeletal muscle tissue to the anabolic properties of amino acids for at least 24 hours [64]. This enhanced sensitivity is of great relevance, suggesting that, as opposed to aging per se, anabolic resistance to protein intake may be more related to the level of physical activity of the specific individual. In keeping with this idea, we [65] and others [66] have demonstrated that 2 weeks of inactivity (muscle disuse via limb immobilization), or even simply decreased physical activity (reduced step count) [67], can also induce anabolic resistance to amino acid administration or protein ingestion. This opens up the attractive possibility that an active older person can successfully overcome anabolic resistance on a daily basis and therefore attenuate age-related muscle loss. Moreover, given the relatively long-lasting effects of physical activity upon muscle protein metabolism [64], it is important to consider the interaction between nutrition and exercise and address their synergistic effects on muscle protein turnover within the context of active elderly individuals. Such information will help to define the optimal protein requirements for healthy, active elderly persons to support the maintenance or increase in muscle mass, strength and physical performance.

## Dietary Protein to Support Post-Exercise Muscle Protein Accretion

Aside from food intake, muscle contraction represents the main physiological stimulus controlling muscle protein turnover. Following a single bout of exercise, muscle protein synthesis rates are increased within 2–4 hours, an effect which persists for up to 16 hours in trained [68] and 24–48 hours in untrained [68–70] individuals. A single bout of exercise also increases muscle protein breakdown rates [70, 71], albeit to a lesser extent than the increase in protein synthesis rates. As such, exercise improves the net muscle protein balance; an effect that is likely maintained for up to 48 hours [70]. However, in the absence of nutrient intake, muscle protein balance will still remain negative [70]. For these reasons, it has often been suggested that the timing of protein or amino acid provision with respect to physical activity and exercise is instrumental in modulating the overall anabolic response [72, 73].

The ingestion of dietary protein in close temporal proximity to physical activity and/or exercise has a synergistic impact on muscle protein synthesis rates, such that greater muscle protein synthesis rates are observed when compared with settings where a single stimulus is provided [52, 74–76]. Furthermore, the modest post-prandial hyperinsulinemia that occurs following dietary protein ingestion may be of sufficient magnitude to maximally inhibit post-exercise muscle protein breakdown rates [29, 77, 78]. The resultant improvement in muscle protein balance following the combined effects of exercise and nutrition allows net muscle protein accretion during recovery from exercise. It has generally been accepted that muscle protein breakdown rates are less responsive to exercise and/or nutritional stimuli when compared with muscle protein synthesis rates, and therefore changes in muscle protein synthesis rates are presumed to have a greater influence over long-term changes in muscle mass [70, 79]. It is apparent that dietary protein ingestion prior to [73, 80], during [81, 82], immediately after [52, 76, 83, 84] or 4 hours [84] after exercise all elicit comparable increases in post-exercise muscle protein synthesis rates. Consequently, a specific and narrow ‘window of opportunity’ may not be as vital as often suggested, but protein consumption in close temporal proximity to physical activity clearly forms an important recommendation to maximize the skeletal muscle adaptive response. It is also true, as mentioned above, that a single bout of exercise can elevate muscle protein synthesis rates for up to 48 hours [68–70] and it is known that skeletal muscle retains its exercise-induced enhanced sensitivity to the anabolic properties of amino acids for at least 24 hours following physical activity [64]. This suggests that the older individual may gain an additional benefit (from a muscle protein synthetic perspective) from all meals consumed within 1–2 days following a single bout of exercise (rather than solely the post-exercise meal). It is important to acknowledge, however, that at present no data are available concerning how post-exercise meals may influence the anabolic response to consecutive meals consumed thereafter. Nevertheless, this information brings to light the importance of carefully considering nutrition over the course of the day and not exclusively within the immediate temporal proximity of a single bout of exercise.

In general, ingestion of a protein-rich meal results in net muscle protein accretion for a period of ~4 hours [74], with peak stimulation of post-prandial muscle protein synthesis rates occurring at ~2 hours following meal ingestion [74]. Based on this, current guidelines for young athletes wishing to maximize gains in muscle mass and strength during resistance-type exercise training programmes generally advise the consumption of 4–6 smaller, high protein meals per day [83]. The same rationale can be applied to the active older adult, particularly in the 24–48 hours following physical activity where the benefits from each meal would likely be enhanced. An emerging strategy to increase meal frequency and promote improved 24-hour muscle protein synthesis rates is to consider the overnight period. We have shown that the low muscle protein synthesis rates observed nocturnally [85] can be robustly stimulated by protein ingestion prior to sleep [86] or by intra-gastric protein administration during sleep [85] in young or elderly men. In keeping with the concept that frequent meals may optimize post-exercise reconditioning, recent evidence reports that, following a bout of physical activity, four smaller protein meals (20 g) spread across the day resulted in greater 12-hour muscle protein synthesis rates compared with larger (40 g) protein meals consumed on two occasions [87]. Moreover, the same study also demonstrated that the same amount of protein consumed in 10 g doses on eight occasions was less effective at increasing post-exercise muscle protein synthesis rates compared with four boluses of 20 g [87]. These data underline the importance of also considering optimal protein sources and amounts to maximize the benefits of each meal during the post-exercise phase.

## Type and Amount of Dietary Protein

Aside from considerations of the specific timing of nutrition in relation to physical activity, research has also investigated the individual properties of a meal that can maximize the anabolic response to food intake. As discussed, protein is the fundamentally anabolic macronutrient, hence studies have attempted to define the optimal amount [83, 88–90] and type [91–94] of dietary protein to maximize muscle protein synthesis rates. Other studies have looked at the impact of co-ingesting other macronutrients with protein [95–97] or specific supplementation with other compounds [98–100]. The muscle protein synthetic response to dietary protein ingestion increases in a dose-response fashion [83, 88–90], with maximal post-exercise muscle protein synthesis rates being achieved in young men following the ingestion of ~20 g of a high quality dietary protein [83, 88, 90]. However, in elderly individuals, more protein may be required to maximize post-prandial muscle protein synthesis rates. In support, ingestion of 35 g whey protein increases resting muscle protein synthesis rates to a greater extent than 20 g [89]. It has also been shown that ingestion of 40 g whey protein results in greater post-exercise muscle protein synthesis rates when compared with the ingestion of 20 g whey protein in older men [88]. Highlighting the importance of paying close attention to protein dose, a recent study reported that when ample protein is consumed across all meals (~30 g per meal), a favourable 24-hour muscle protein synthetic rate is achieved when compared with the consumption of the same amount of protein unevenly distributed over the various meals (10–60 g per meal) [101]. Interestingly, when extreme skewed diets are employed (~80 % of daily protein intake [~80–85 g] consumed in a single meal with the rest spread across the remaining meals), favourable effects on nitrogen retention, whole body protein balance and/or lean mass have been reported compared with a more spread feeding approach [102, 103]. However, in these studies, the spread feeding condition provided protein amounts per meal generally below the likely requirement for optimal stimulation of muscle protein synthesis rates in elderly individuals. Moreover, to date, such studies have not investigated the impact of these severe modifications of protein amounts consumed throughout the day on muscle protein turnover. Therefore, taken together, available data illustrate the importance of consuming ample protein with each main meal, avoiding unnecessarily high protein meals (as they seem to infer little to no additional benefit and result in a ‘waste’ of daily protein intake) and avoiding the typically low protein breakfast often reported in older populations [104]. It is worthy to note, however, that it is currently unknown whether 40 g of protein represents an upper limit in terms of what can be utilized by an elderly individual for muscle protein synthesis, especially in the post-exercise phase [105]. Likewise, how the type, duration and intensity of exercise performed by the individual may affect a dose–response relationship in the rates of synthesis of different fractions of skeletal muscle proteins also remains to be investigated. As such, available information thus far suggests that the ingestion of 30–40 g of a high quality dietary protein immediately following physical activity and at regular intervals thereafter may best support reconditioning in the active older adult.

Theoretically, when ample protein is provided following exercise, it is likely that the type and composition of the protein is less relevant since the sensitivity of the skeletal muscle tissue to the anabolic properties of amino acids is elevated during recovery from exercise [106]. However, for the active older adult wishing to maximize muscle protein anabolism, consuming large and frequent protein-rich meals can be arduous. Accordingly, scientific attention has also turned to the specific type of dietary protein capable of best promoting post-prandial anabolism to a given dose [27, 51, 91, 92, 107]. The key properties determining the anabolic potential of a dietary protein appear to be its digestion and absorption kinetics, and its amino acid composition. A dietary protein exhibiting rapid digestion and subsequent absorption kinetics, such as whey protein, has been shown to elicit a greater muscle protein synthetic response compared with ‘slower’ proteins such as soy [92, 108] or casein [51, 92] in young and older men. Moreover, even if a slow protein such as casein is artificially pre-digested (hydrolyzed) to ensure digestion and absorption kinetics similar to whey, the post-prandial muscle protein synthetic response of casein is still less pronounced compared with whey [51]. The latter may be attributed to the greater leucine content in whey compared with casein protein [27, 51]. Indeed, it has been shown that fortifying a single bolus of dietary protein with only 2–3 g of crystalline leucine can amplify the post-prandial anabolic response in elderly men [27, 28]. Consequently, it appears that selecting a rapidly digestible protein source, with a high leucine content (or fortifying other dietary protein sources with leucine) represents a feasible strategy to reduce the protein dose required for optimal post-prandial muscle protein synthesis rates. However, more work is necessary to assess the relevance of such a strategy in a post-exercise recovery setting in older adults.

Recent work has started to address whether co-ingestion of other macronutrients with protein modulates the post-prandial muscle protein synthetic response. For instance, carbohydrate co-ingestion could increase post-prandial muscle protein synthesis rates by increasing circulating insulin in the older (less insulin-sensitive) population. However, we [96, 97] and others [95] have been unable to observe any benefits of carbohydrate co-ingestion with protein in either young or older men at rest or following exercise. The potential impact of dietary fat on the anabolic response to meal ingestion is less clear, as few data are currently available [109], particularly obtained within a nutritionally relevant context [110]. Nonetheless, at this stage we can state that frequent meals consumed by older adults in close proximity to exercise do not need to be of a high energy density; rather, attention should be focussed on the amount and type of dietary protein contained within each meal.

**Fig. 1:**
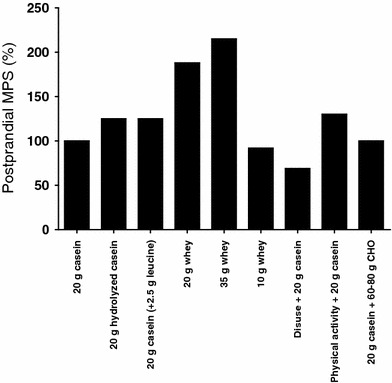
Schematic representation of the relative muscle protein synthetic (MPS) response to the ingestion of a single protein bolus in elderly individuals. A 20 g dose of casein protein is taken as a control/normal response due to its resemblance to the protein content found in a ‘normal meal’ (in terms of protein quantity, digestion and absorption kinetics, and amino acid profile) and is given an arbitrary ‘100 %’ value. Thereafter, bars refer to the relative modulations (either increase or decrease) from ‘normal’ (i.e., 100 %) by different interventions, details of which are reported on the *x* axis. Data were extrapolated from numerous studies performed within our laboratory [27, 51, 52, 65, 89, 96, 97]. Note: wherever possible, the 100 % control response for each intervention is obtained from the values obtained within that specific study. *CHO* carbohydrate

The current understanding of nutritional/physical activity manipulations capable of manipulating the post-prandial muscle protein synthetic response in elderly individuals is summarized graphically in Fig. 1.

## Dietary Protein to Support Muscle Reconditioning During Exercise Training

Insight into the processes that underlie the regulation of skeletal muscle mass is paramount if we are to understand how healthy, active older adults can maintain or gain skeletal muscle mass. However, ultimately, the impact of nutritional and/or exercise interventions to stimulate muscle protein accretion must be translated to studies assessing clinically relevant end-points, such as changes in skeletal muscle mass, body composition, muscle strength, and functional capacity. Habitual protein consumption generally declines with advancing age [111]. Importantly, a higher (>1.0 g per kg body mass per day) dietary protein intake has been associated with an attenuated decline in muscle mass and functional performance in older adults in both epidemiological and intervention-based studies [112–114]. Moreover, prolonged resistance-type exercise training has been shown to represent the most effective strategy to increase muscle mass and strength, as well as functional capacity in the elderly. Various long-term intervention studies have confirmed the paradigm that protein feeding in close temporal proximity to each bout of exercise augments training-induced gains in muscle mass and strength [20–24, 115–120]. However, many other studies have been unable to confirm these surplus benefits of protein supplementation above and beyond those afforded by exercise training only [121–129]. We previously argued that small cohorts and differences in study design may explain this apparent discrepancy in the literature [17]. In order to resolve this issue, we recently conducted a meta-analysis to more thoroughly examine the proposed benefits of protein supplementation to further augment the adaptive response during prolonged resistance-type exercise training in young and older men [17]. This approach revealed that protein supplementation augmented training-induced gains in muscle mass and strength in adults of all ages. Older individuals who ingested dietary protein around the time of each exercise bout exhibited a 38 and 33 % greater gain in fat-free mass and strength, respectively, compared with those not consuming additional protein with training [17]. Interestingly, we have recently shown that additional dietary protein supplementation was required to actually gain additional muscle mass at all during prolonged resistance-type exercise training in frail, elderly subjects [130]. During the 6-month intervention, the frail elders were unable to gain measurable increases in skeletal muscle when no additional protein was provided. Taken together, these data illustrate the importance of dietary protein to support muscle reconditioning for the active older adult. However, given the variance in findings from smaller cohort studies, we feel that the surplus benefits of dietary protein supplementation largely depend on the type, dose and timing of the protein supplements that are being consumed.

## Nutritional Compounds to Support Active Aging

Although a healthy, well balanced diet forms the foundation of any nutritional plan to support muscle reconditioning, there may be nutritional compounds that could help to enhance the muscle adaptive response in the active older adult. Oral supplementation with creatine has been a long-standing, evidenced-based strategy employed by recreational and competitive athletes as an ergogenic aid for high-intensity exercise performance, and to augment training adaptations in young athletes [131–133]. Creatine is generally consumed in relatively large doses (~20 g per day) for 5–7 days in order to ‘load’ the muscle [134], with a maintenance dose of 2–5 g per day thereafter to maintain elevated muscle creatine levels for several weeks [131–133]. Ergogenic benefits are attributed to an increased muscle store of phosphocreatine, allowing improved adenosine triphosphate delivery from phosphocreatine hydrolysis during high-intensity exercise, allowing for a greater training stimulus and, ultimately, an augmented training adaptation. Although most studies have been conducted in young athletes, a recent meta-analysis concluded that the addition of oral creatine supplementation to regular exercise training in older adults also allows for greater gains in muscle mass and strength [100]. It seems evident that this would be of more relevance to the more experienced older athletes, who may have reached limits in the exercise-induced gains in muscle mass and strength, compared with that of the novice healthy adult starting out on an exercise intervention program.

Another nutritional approach that has received much recent attention is the fish oil-derived omega-3 fatty acids (eicosapentaenoic acid, EPA; docosahexaenoic acid, DHA) [98, 99, 135–138]. It has been reported that 8 weeks of omega-3 supplementation (4 g per day) results in a greater muscle protein synthetic response to amino acid administration in adults of all ages [98, 99]. Importantly, this appears to translate into functional benefits. Long-term (6 months) fish oil supplementation has been reported to improve physical performance indices such as walking speed [138]. Moreover, during a 90-day resistance-type exercise training program in older women, the consumption of fish oil supplements (2 g per day) resulted in greater gains in muscle strength and functional capacity when compared with a placebo [137]. More data are required to determine whether fish oil supplementation represents an effective adjunct strategy to augment the exercise-induced gains in muscle mass, strength and functional performance in the older population.

## Conclusions

Developing nutritional strategies to support skeletal muscle reconditioning in active older adults is of great clinical relevance given our rapidly aging population. Prior work investigating the mechanisms of age-related muscle loss has highlighted a reduced muscle protein synthetic response to dietary protein ingestion in older individuals as a key responsible factor. However, performing physical activity and consuming dietary protein in close temporal proximity can compensate for anabolic resistance by increasing the muscle protein synthetic response to food intake. Evidence suggests that 30–40 g of protein represents an optimal dose to best promote (post-exercise) muscle protein synthesis rates in older adults. Moreover, sub-optimal doses of dietary protein can be optimized by selecting proteins with rapid digestion and absorption kinetics that are also rich in leucine (e.g., whey), and/or specifically enriched protein with leucine. Long-term intervention studies have been able to demonstrate the surplus benefits of dietary protein supplementation to further augment the gains in muscle mass and strength during prolonged resistance-type exercise training. Emerging evidence also suggests that oral creatine and/or fish oil-derived fatty acid supplementation may be effective as a means to enhance training adaptations in the older population. Future work will translate the mechanistic insight of how the timing, amount and type of dietary protein impacts the muscle protein synthetic response to more effective interventional strategies to maximize gains in muscle mass, strength and function during exercise training in the older population. Such studies will allow a further refinement of dietary protein recommendations to support active aging.
